# Impaired lung regeneration after SARS‐CoV‐2 infection

**DOI:** 10.1111/cpr.12927

**Published:** 2020-10-20

**Authors:** Hongxia Shao, Zhonghua Qin, Bei Geng, Junping Wu, Lixia Zhang, Qiuyang Zhang, Qi Wu, Li Li, Huaiyong Chen

**Affiliations:** ^1^ Department of Respiratory Medicine Haihe Clinical College of Tianjin Medical University Tianjin China; ^2^ Department of Laboratory Medicine Haihe Clinical College of Tianjin Medical University Tianjin China; ^3^ Department of Basic Medicine Haihe Clinical College of Tianjin Medical University Tianjin China; ^4^ Department of Tuberculosis Haihe Clinical College of Tianjin Medical University Tianjin China; ^5^ Key Research Laboratory for Infectious Disease Prevention for State Administration of Traditional Chinese Medicine Tianjin Institute of Respiratory Diseases Tianjin China; ^6^ Department of Basic Medicine Haihe Hospital Tianjin University Tianjin China; ^7^ Tianjin Key Laboratory of Lung Regenerative Medicine Tianjin China

To the Editor,

The 2019 coronavirus disease (COVID‐19), caused by severe acute respiratory syndrome coronavirus 2 (SARS‐CoV‐2), has become a global pandemic. Until 13 September 2020, SARS‐CoV‐2 caused more than twenty eight million infections and nine hundred thousand deaths worldwide. SARS‐CoV‐2 uses angiotensin‐converting enzyme 2 (ACE2) for cell entry into various lung cells including endothelial cells and alveolar type 2 (AT2) epithelial stem cells.^1^, ^2^ As cell damage usually results in the release of cell‐specific proteins into the circulation, these proteins can be detected to evaluate cellular injury.^3^ So far, there is little information on cellular repair in the lung after the clearance of SARS‐CoV‐2. In this case series, we assayed serum levels of surfactant D (SPD; AT2 cell marker), the receptor for advanced glycation products (RAGE; AT1 cell marker), von Willebrand factor (vWF; endothelial cell marker), and laminin (lung matrix) in patients with COVID‐19 at acute infection stage (9‐14 days after illness onset) and recovery stage (14 days after virus clearance) admitted to Haihe Hospital, Tianjin University in China.

In this study, COVID‐19 patients with confirmed SARS‐CoV‐2 infection by polymerase chain reaction were admitted to Haihe Hospital, Tianjin University, from 21 January to 20 March 2020. Subjects were classified using the following criteria: 1) moderate cases: fever and respiratory symptoms with radiological findings of pneumonia; 2) severe cases: meeting any one of the following criteria: respiratory distress, respiratory rate ≥30 bpm, hypoxia (Spo_2_ ≤ 93%) or PaO_2_/FiO_2_ ≤ 300 mm Hg. Thirty sex‐ and age‐matched health workers who completed physical examination between 17 June 2020 and 24 June 2020 at our hospital were included as healthy controls. Sera were collected for measurements of SPD, RAGE, laminin and vWF by an enzyme‐linked immunosorbent assay. The study was approved by Haihe Hospital Ethics Committee, and written consent was obtained from the patients before enrolment. The chi‐square test was used to compare proportions for categorical variables, while independent group *t* tests or the Mann‐Whitney test was used to compare continuous variables.

The study population included 115 hospitalized COVID‐19 patients and 30 healthy controls. For healthy controls, the median age was 51 years (IQR, 44‐55; range, 27‐59 years), and 15 (50%) were men. The median age of included COVID‐19 patients was 49 years (IQR, 37‐62; range, 19‐91 years), and 60 (52%) were men (Table S1). Hypertension and diabetes were the most common coexisting conditions. Most common symptoms during clinical course included fever (87 [76%]), cough (58 [51%]) and expectoration (40 [35%]). Severe patients were more prone to high fever than moderate patients (Table S1). Compared to moderate patients, the severe patients displayed lower lymphocyte count and sodium levels, but higher levels of creatinine and creatinine kinase (Table S1).

The serum levels of SPD, RAGE and vWF were significantly increased in COVID‐19 patients at acute infection stage (Figure 1A). Serum RAGE and vWF levels decreased, but the SPD level continued to increase at recovery stage. Serum laminin level was not boosted until recovery stage. We tracked serum levels of all these four cellular markers in 14 COVID‐19 patients at both the acute infection stage and recovery stage, and a similar trend was observed (Figure 1B). Elevation of serum SPD through acute to recovery stage was found in both moderate and severe male COVID‐19 patients (Figure 1C). However, such elevation of serum SPD was not seen in either moderate or severe female COVID‐19 patients (Figure 1D). Similar to SPD, serum laminin level was increased from acute to recovery stage in both moderate and severe groups of male COVID‐19 patients (Figure 1C). Such increase was observed in moderate but not severe female COVID‐19 patients (Figure 1D). In contrast, serum levels of RAGE and vWF showed an opposite trend in both moderate and severe male COVID‐19 patients through acute to recovery stage (Figure 1C). For female COVID‐19 patients, moderate but not severe group exhibited a reduction of serum levels of RAGE and vWF through acute to recovery stage (Figure ID).

**Figure 1:**
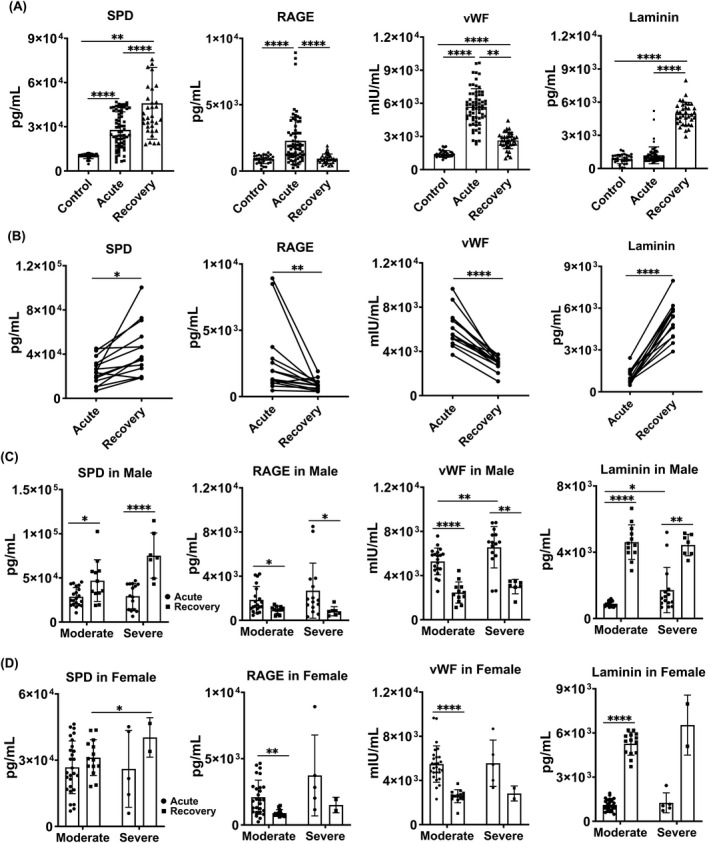
Cellular injury in the lung of COVID‐19 patients. A, Serum levels of SPD, RAGE, laminin and vWF were measured in healthy subjects, COVID‐19 patients at acute infection stage (9‐14 days after SARS‐CoV‐2 infection) and COVID‐19 patients at recovery stage (14 days after SARS‐CoV‐2 clearance) by ELISA. B, Levels of injury markers in 14 patients at acute infection stage and recovery stage. C, Levels of injury markers in male moderate and severe patients at acute infection stage and recovery stage. D, Levels of injury markers in female moderate and severe patients at acute infection stage and recovery stage. Data represent the median ± SD *P* values were determined with unpaired, two‐sided Mann‐Whitney *U* test. *P < .05; **P < .01; ****P < .0001. All statistical analyses were performed using SPSS version 19.0 software

This study represents the first evaluation of cellular injury associated with SARS‐CoV‐2 infection. The illness severity and fatality of the disease are associated with SARS‐CoV‐2‐induced inflammatory storm. The resulting tissue damage must be recovered soon to avoid fibrosis progression even after the virus is cleared.^4^ We found that SARS‐CoV‐2 infection caused damage to AT2, AT1, endothelial cells and lung structures. This was consistent with a previous report that alveolar cells and lung structure were severely damaged during infection stage based on biopsy analysis of two COVID‐19 patients.^5^ Immunofluorescent analysis of lungs of five deceased patients suggested that survived AT2 cells were also shown to initiate repair mechanism after SARS‐induced lung damage.^6^ But there has been few study evaluating the capacity of lung regeneration in recovered COVID‐19 patients. Through analysing serum cellular markers in 115 COVID‐19 patients, we for the first time provide evidence to suggest that damage to AT1 cells and endothelial cells was no longer evident at the recovery stage after virus clearance. However, damage to AT2 cells and lung structures still persisted two weeks after SARS‐CoV‐2 was removed. And such injury was more obvious in both moderate and severe male patients than female patients. The limitations of this work include the small number of COVID‐19 patients from a single centre. Nonetheless, AT2 cells serve as stem cells for repairing alveolar gas‐exchanging epithelium.^7^ Our study, therefore, suggests that insufficient alveolar repair may increase lung vulnerability to inhaled microbes and substances or lead to lung fibrosis especially in discharged male COVID‐19 patients. Therefore, our data demonstrate that it will take an unexpectedly longer time to recover from SARS‐CoV‐2 infection, which is worth further clinical investigation.^8^

## CONFLICT OF INTEREST

All authors declared no conflict of interest.

## AUTHORS’ CONTRIBUTION

HS and BG had full access to the clinical data and took responsibility for the integrity of the data; ZQ performed the experiments; JW. LZ and QZ assisted the experiments; HC, HS, ZQ, QZ, BG and LL analysed and interpreted the data; HC, QW and LL designed the study; HC drafted the manuscript; All authors edited and approved the manuscript.

### DATA AVAILABILITY STATEMENT

The authors declare that all the data supporting the findings of this study are available within the article and its Supplementary Information files and from the corresponding authors on reasonable request.

## Supporting information

Table S1
