# Supplementary material for: Impaired lung regeneration after SARS‐CoV‐2 infection
Source: Cell Prolif. 2020 Oct 20;53(12):e12927. doi: 10.1111/cpr.12927 (PMC7645888; doi:10.1111/cpr.12927)
Supplement: Supplementary file 1 — Table S1 [file CPR-53-e12927-s001.docx]

**Table S1. Characteristics, immunological indicators, treatments and clinical course of patients with COVID-19**

|  | **Total (n=115)** | **Moderate (n=79)** | **Severe (n=36)** | **p-value**^a^ | |
| --- | --- | --- | --- | --- | --- |
| **Characteristics** |  |  |  |  | |
| Age, median (IQR), y | 49 (37-62) | 48 (36-60) | 55 (42-65) | 0.107 | |
| Sex |  |  |  |  | |
| Male | 60(52) | 34 (43) | 26 (72) |  | |
| Female | 55 (49) | 45 (57) | 10 (28) |  | |
| Exposure history in Wuhan >2weeks | 17 (15) | 11 (14) | 6 (17) | 0.779 | |
| Familiar cluster | 67 (58) | 48 (61) | 19 (49) | 0.541 | |
| Coexisting conditions |  |  |  |  | |
| Any | 36 (31) | 25 (32) | 11 (31) | 1.000 | |
| Hypertension | 24 (21) | 16 (19) | 9 (25) | 0.468 | |
| Diabetes | 12 (10) | 8 (10) | 4 (11) | 1.000 | |
| COPD | 2 (2) | 2 (3) | 0 (0) | 1.000 | |
| Liver disease | 3 (3) | 3(4) | 0 (1) | 0.245 | |
| **Signs and symptoms** |  |  |  |  | |
| Fever | 87 (76) | 55 (70) | 32 (89) | 0.034* | |
| Cough | 58 (51) | 39 (49) | 19 (53) | 0.841 | |
| Myalgia or fatigue | 17 (15) | 10 (13) | 7 (19) | 0.339 | |
| Expectoration | 40 (35) | 29 (37) | 11 (31) | 0.673 | |
| Headache | 5 (5) | 3 (4) | 2 (6) | 0.648 | |
| Diarrhea | 7 (6) | 6 (8) | 1 (3) | 0.431 | |
| Respiratory rate > 24 breaths/min | 15 (13) | 9 (11) | 6 (17) | 0.551 | |
| Arterial oxygen pressure (mm Hg) | 98 (98-100) | 98 (98-100) | 98 (96-100) | 0.627 | |
| PO_2_/FiO_2_ | 397 (307-504) | 429 (347-541) | 317 (286-434) | 0.003* | |
| Mean arterial pressure (mm Hg) | 96 (88-105) | 94 (88-104) | 97 (88-106) | 0.971 | |
| **Laboratory examination at admission (median (IQR))** | | | | |  |
| White blood cell count  (x10^9^/L, reference range: 4-10) | 4.7 (3.7-5.9) | 4.7 (4.0-6.1) | 4.7 (3.6-5.5) | 0.677 | |
| Neutrophil count  (x10^9^/L, reference range: 2-7.5) | 3.1 (2.3-4.0) | 3.0 (2.3-3.8) | 3.2 (2.4-4.5) | 0.675 | |
| Lymphocyte count  (x10^9^/L, reference range: 0.8-4) | 1.0 (0.7-1.4) | 1.1 (0.8-1.5) | 0.8 (0.6-1.2) | 0.014* | |
| Haemoglobin  (g/L, reference range: 120-160) | 133 (126-149) | 132 (126-146) | 137 (128-154) | 0.120 | |
| Platelet count  (x10^9^/L, reference range: 100-400) | 178 (140-219) | 186 (148-217) | 167 (126-223) | 0.150 | |
| D-dimer  (mg/L, reference range: 0-0.55) | 0.5 (0.3-0.8) | 0.5 (0.3-0.8) | 0.4 (0.2-0.8) | 0.362 | |
| Alanine aminotransferase  (U/L, reference range: 9-52) | 35 (26-48) | 35 (26-47) | 40 (25-62) | 0.526 | |
| Aspartate aminotransferase  (U/L, reference range: 14-36) | 30 (23-38) | 28.5 (23-35) | 31 (24-59) | 0.104 | |
| Potassium  (mmol/L, reference range: 3.5-5.1) | 4.0 (3.7-4.4) | 3.9 (3.7-4.3) | 4.2 (3.8-4.5) | 0.146 | |
| Sodium  (mmol/L, reference range: 137-145) | 138 (136-140) | 139 (137-140) | 137 (135-139) | 0.001* | |
| Creatinine  (μmol/L, reference range: 58-110) | 56 (46-73) | 56 (44-68) | 66 (49-85) | 0.029* | |
| Creatinine kinase  (U/L, reference range: 55-170) | 64 (41-104) | 56 (38-92) | 83 (53-193) | 0.015* | |
| Lactate dehydrogenase  (U/L, reference range: 313-618) | 488 (399-622) | 483 (408-579) | 505 (251-745) | 0.636 | |
| **Chest Radiology** |  |  |  |  | |
| Bilateral involvement on chest radiographs | 95 (83) | 61 (77) | 34 (94) | 0.032* | |
| Pneumonia | 112 (97) | 77 (98) | 35 (97) | 1.000 | |
| **Clinical treatments and outcomes** | 1 (1) | 0 (0) | 1 (3) |  | |
| Admission to intensive care unit | 1 (1) | 0 (0) | 1 (3) |  | |
| Acute respiratory distress syndrome |  |  |  |  | |
| Treatment |  |  |  |  | |
| Interferon alpha inhalation | 2 (2) | 2 (3) | 0 (0) |  | |
| Arbidol+interferon alpha inhalation | 6 (5) | 4 (5) | 2 (6) |  | |
| Lopinavir/ritonavir+Interferon alpha inhalation | 20 (17) | 18 (23) | 2 (6) |  | |
| Arbidol+Lopinavir/ritonavir | 1 (1) | 1 (1) | 0 (0) |  | |
| Arbidol+Lopinavir/ritonavir+Interferon alpha inhalation | 86 (75) | 54 (68) | 32 (89) |  | |
| Antibiotics | 57 (50) | 31 (39) | 26 (72) |  | |
| Corticosteroid and gamma globin | 29 (25) | 14 (18) | 15 (42) |  | |
| Chinese traditional medicine | 118 (100) | 82 (100) | 36 (100) |  | |
| Prognosis |  |  |  |  | |
| Discharge | 115 (100) | 79 (100) | 36 (100) |  | |

The χ2 test was used to compare proportions for categorical variables presented as number (% of total subjects). Independent group t tests or the Mann-Whitney test was used to compare continuous variables presented as median (interquartile range, IQR).

^a^ *P* values indicate difference between moderate and severe patients. *P* < 0.05 was considered statistically significant.
